# Helminth immunoregulation: The role of parasite secreted proteins in modulating host immunity

**DOI:** 10.1016/j.molbiopara.2009.04.008

**Published:** 2009-09

**Authors:** James P. Hewitson, John R. Grainger, Rick M. Maizels

**Affiliations:** Centre for Immunity, Infection and Evolution, Institute of Immunology and Infection Research, University of Edinburgh, West Mains Road, Edinburgh EH9 3JT, UK

**Keywords:** Antioxidant, Cystatin, Cytokine, Helminth, Immune evasion, Lectin, Protease, Serpin

## Abstract

Helminths are masterful immunoregulators. A characteristic feature of helminth infection is a Th2-dominated immune response, but stimulation of immunoregulatory cell populations, such as regulatory T cells and alternatively activated macrophages, is equally common. Typically, Th1/17 immunity is blocked and productive effector responses are muted, allowing survival of the parasite in a “modified Th2” environment. Drug treatment to clear the worms reverses the immunoregulatory effects, indicating that a state of active suppression is maintained by the parasite. Hence, research has focussed on “excretory–secretory” products released by live parasites, which can interfere with every aspect of host immunity from initial recognition to end-stage effector mechanisms. In this review, we survey our knowledge of helminth secreted molecules, and summarise current understanding of the growing number of individual helminth mediators that have been shown to target key receptors or pathways in the mammalian immune system.

## 1 Immune modulation during helminth infection

The capacity of helminth parasites to modulate the immune system underpins their longevity in the mammalian host [1,2]. There is consequently intense interest in understanding the molecular basis of helminth immunomodulation [3,4]. The remarkable range of parasite life histories, transmission strategies, and physiological niches, is reflected in the variety of immunomodulatory activities observed across the three taxonomic categories (nematodes, cestodes, and trematodes) that comprise the helminth grouping [5–9]. However, general patterns have emerged, revealing the ways in which helminths can dampen host immunity, and how immunopathology may result from a dysregulated response to infection [10]. For instance, both schistosome (for example, *Schistosoma mansoni*) and filarial (e.g. *Brugia malayi*) infections result in antigen-specific unresponsiveness in the peripheral T cell populations of heavily infected patients [11–13]. Moreover, helminth infection is associated with diminished reactivity to bystander allergens and autoantigens, both in model systems [8,14] and in human studies [15,16].

A key feature is that helminth immune suppression is dependent on live parasites, as shown *in vivo* by the recovery of responsiveness following curative chemotherapy [17], as well as by the regulatory effects of live parasites *in vitro* [18]. Hence, there is a particular focus on mediators released by live parasites and the analysis of how these products, in total and as individual components, may be responsible for the noted ability of helminths to redirect the host immune system.

## 2 Helminth secreted products: the rationale

Mechanistically, parasite modulation of the immune system is most likely to be effected through the release of soluble mediators which ligate, degrade or otherwise interact with host immune cells and molecules [19]. Modulation may also occur through the release (and death of some proportion) of transmission stages such as the eggs of schistosomes or the newborn microfilarial larvae of filarial parasites. In tissue-dwelling parasites, important engagements also occur at the surface of the helminth itself. Much of the earlier literature on immunological effects of helminth products depended on crude extracts (such as SEA schistosome egg antigen), although the degree to which the host is exposed to constituent molecules was uncertain. While both somatically derived and secreted products are known to have immunological activity [4], the secreted helminth modulators are those most likely to be physiological actors at the interface between live parasites and the host, and these are the subject of this review.

“Excretory/secretory” (ES) is inevitably a working definition, with an imprecise line between products actively exported through secretory pathways and those which may diffuse or leak from the parasite soma. *In vivo*, “secreted” antigens will include digestive enzymes emanating from the intestine of adult worms, as well as uterine contents which female worms release along with transmission stage eggs or larvae. However, parasites may well have adapted such “secretions” to fulfill a new role in the host, once they are released from their primary locale within the worm. Hence, it is rational to analyse all ES products without prejudice as to their physiological origin, and subject them to a full range of biochemical, immunological and proteomic analyses.

Biochemical analyses have primarily concerned enzymatic activities in helminth ES, such as the proteases ranging in activity from parasite invasion [20] to degradation of host chemokines [21]. Where enzymes (also including antioxidants, acetylcholinesterases and platelet activating factor hydrolase) act in an immunological context, these are detailed further in Section 4.7 below. Immunological assays of ES have included the induction of Th2 responsiveness, leading in the case of *S. mansoni* to the products described in Section 4.1. An alternative, transcriptomic-based, avenue led to identifying ES products which are encoded by abundant mRNA species (e.g. filarial ALT proteins [22], see Section 4.9 below). More recently, with the development of helminth genomics, systematic proteomic analyses of many major helminth ES products have become possible (Table 1). These studies revealed a common set of proteins secreted by helminths, including proteases, protease inhibitors, venom allergen homologues, glycolytic enzymes and lectins. However, the relative abundance of each of these varied between different parasites and individual life cycle stage, reflecting the range of sites of parasitism.

Available parasitic helminth genomes encode >10,000 genes [23], a figure supported by independent transcriptomic analyses [24,25]. Bioinformatic approaches to predict secreted proteins on the basis of signal peptide sequences [26,27] have some merit, but in a metazoan not all secretory proteins will be exported from the organism, and proteomic data show a surprisingly large proportion of ES proteins are not encoded with a signal peptide [28–30]; hence empirical proteomic studies remain essential. Although ES products will only represent a fraction of the full genomic complement, determining the function of several hundred secreted proteins is a formidable task involving cloning and recombinant expression, as well as the production of neutralising antibodies.

**Fig. 1:**
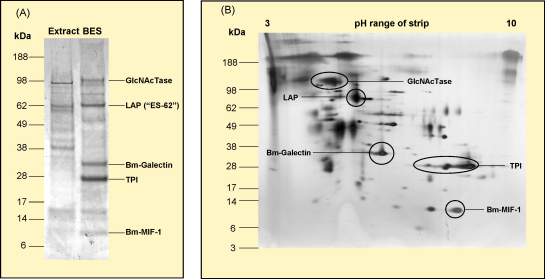
Helminth ES proteins: an example of the complexity of secreted proteins, from adult *B. malayi*[29], highlighting products discussed in the text. (A) One-dimensional gel, Coomassie Blue stained, showing selective secretion compared to whole somatic extract, indicating the migration of *N*-acetylglucosaminyltransferase (GlcNaTase), leucyl aminopeptidase (LAP, the homologue of ES-62), galectin, triose phosphate isomerase (TPI) and *B. malayi* homologue of macrophage migration inhibitory factor-1 (Bm-MIF-1). (B) Two-dimensional, silver stained gel, with the positions of the same proteins indicated.

Several other caveats about our current technologies should be borne in mind. While proteomic analysis can reveal the composition of helminth secretions and the relative abundance of each protein, it gives no information on the non-protein components (e.g. carbohydrates [31,32]), and post-translational modifications are not easily ascertained. Secondly, not all secreted products are macromolecules: filarial parasites secrete prostacyclin and prostaglandin for example [33], and schistosome eggs release free glycans [34]. Thirdly, while proteomic techniques allow unbiased identification of the more abundant ES proteins (Fig. 1), they may still miss those expressed at low, but bioactive, levels [29,30,35]. Even with these reservations in mind, however, it is clear that a rich and fascinating set of parasite modulators have already been discovered.

In the following sections, we briefly summarise in Section 3 the molecular and immunological information available on the secreted products from each major helminth species, before discussing in Section 4 the key individual molecular mediators now identified from the ES products of these parasites.

## 3 Functional and molecular analyses of helminth products

### 3.1 Trematodes: *S. mansoni* and *Fasciola hepatica*

Schistosome infections commence when cercariae of this trematode penetrate the vertebrate skin, transforming into schistosomula larvae in the process. Schistosomulae migrate to the lung, mature as adults in the vasculature, and produce eggs which exit through the intestine. Each of these stages is implicated in immune modulation. Larval secretions are also highly immunogenic vaccine targets as passive immunisation with antisera to ES confers around 50% protection against challenge infection [36]. The same skin-stage schistosome ES directs DCs to drive Th2 responses *in vivo* [37]. This ES contains abundant proteases, including several elastases that facilitate parasite skin penetration [38], and can cleave host IgE antibodies [39]. The presence of multiple isoforms of cercarial elastase and a metalloprotease was confirmed by proteomics of cultured parasites [40,41], and by proteomic analysis of human skin traversed by invading cercariae [42]. Additionally, skin-stage parasites were shown to secrete a number of glycolytic enzymes, such as triose phosphate isomerase, GADPH, aldolase and enolase, as well as several homologues of the venom allergen-like (VAL) family, as discussed in Section 4.8. Cercarial ES also contains the immunomodulator Sm16 that can inhibit toll-like receptor signalling in monocytes [43].

Completion of the schistosome life cycle requires that eggs transit from the mesenteric veins, through the intestinal mucosa, into the lumen of the intestine, in a manner dependent on the inflammatory response of the host. Proteomic analysis of egg ES reveals two abundant proteins, alpha-1 (since renamed IPSE, IL-4-inducing principle of schistosome eggs) and a ribonuclease omega-1 [28,44] (see Section 4.1). Glycolytic enzymes (particularly aldolase and enolase) are again well represented in the secretions, as are VAL homologues.

The trematode liver fluke *F. hepatica* releases an extensive series of cathepsin L thiol proteases, which can induce significant protection in vaccine form [45]. Adult flukes also secrete thioredoxin peroxidase, which stimulates the alternative activation of macrophages both *in vitro* and *in vivo* [46]. A recent proteomic analysis of larval *F. hepatica* has identified additional antioxidant enzymes as prominent ES products [47].

### 3.2 *Filarial nematodes*: *B. malayiandAcanthocheilonema viteae*

The immunomodulatory potential of secretions of adult *Brugia* (BES) were noted some years ago, when BES treatment of infected dogs resulted in the loss of antigen-driven lymphocyte proliferation [48]. Further, in mice, BES injection generated suppressive alternatively activated macrophages [49]. Together these studies show that *Brugia* secretions mimic at least some of the immunomodulatory effects of actual infection.

The secretomes of adult and microfilarial stages of *B. malayi* have recently been analysed [29,30], matching data to the recently published genome [23]. Abundant proteins secreted by adult parasites include the cytokine homologue Bm-MIF-1 [50], a leucyl aminopeptidase, the PC-bearing protein *N*-acetylglucosaminyltransferase, and a *Brugia* galectin *Bm*-GAL-1 [29]. Surprisingly, the most abundant protein released by adult parasites, highly enriched compared to worm homogenate, was the glycolytic enzyme triose phosphate isomerase (TPI). TPI is also preferentially secreted by the plant nematode *Meloidogyne incognita* [51], and its role may not therefore be specific to the mammalian immune system. Experimental testing of TPI and the other major ES products are now under way in our laboratory.

*B. malayi* microfilariae secrete qualitatively and quantitatively different proteins to adult parasites, likely reflecting their different location within the host [30]. Abundant proteins include the diagnostic antigen R1 [52], and a serpin (serine protease inhibitor, SPN-2; [53]). Both adults and microfilariae release phosphatidylethanolamine binding protein (homologous to *Onchocerca volvulus* Ov-16 and *Toxocara canis* secreted TES-26 [54]). Secretions from the mosquito-borne infective larval (L3) stage are more difficult to analyse due to limitations on material, although it is known from biochemical studies that a novel protein family (abundant novel transcript, ALT) is released from glandular stockpiles, while other products include cysteine protease inhibitors and a homologue of VAL (B Gregory and J Murray, unpublished observations).

Rodent models for filariasis include *A. viteae*, in which adult worms can be recovered from the peritoneal cavity of gerbils. Adults secrete a single predominant molecule, ES-62, a leucyl aminopeptidase carrying multiple phosphorylcholine (PC) sidechains [55], as discussed in Section 4.2 below.

### 3.3 Rodent intestinal nematodes: *Nippostrongylus brasiliensis* and *Heligmosomoides polygyrus*

*N. brasiliensis* is a widely used model of nematode infection of rodents characterised by robust Th2 differentiation and parasite clearance within a week [56]. *In vivo* administration of *N. brasiliensis* adult ES (NES), directly [57] or through NES-pulsed dendritic cells (DCs) [58], results in strong Th2 responses. NES also induces alternative activation of macrophages [49]. Notably, NES results in strong IL-4 production, even in the presence of Th1/Th17-inducing complete Freund's adjuvant, indicating a dominant Th2-inducing component which is heat- and protease-labile [57,58], but is not itself a protease. As well as driving Th2 responses *in vivo*, NES can also regulate pro-inflammatory Th1 responses, inhibiting both mitogen-dependent interferon-γ production by naïve mesenteric lymph node cells [59] and LPS-induced IL-12p70 production by DC [58]. Notably, NES under the same conditions does not reduce IL-6 production, and heat-inactivated NES has no inhibitory properties, indicating that a selective and heat-sensitive pathway is in play. Blocking IL-12p70 responsiveness is a common property of many helminth ES products, and may represent a shared strategy to forestall Th1 responses [10].

Surprisingly, despite acting as a Th2-inducing adjuvant, NES can also inhibit Th2-mediated pathology. Both *N. brasiliensis* infection [60] and NES alone can inhibit allergen-induced lung inflammation [61]. *In vivo* studies showed that ES from *N. brasiliensis* L3 larvae (L-NES) inhibited LPS-dependent neutrophil recruitment to the lungs [62]. Despite the protective effects of NES against lung inflammation, L-NES is intrinsically allergenic [63], suggesting that different components may be acting in opposing manners over the longer term. Currently, few individual components of NES have been identified (for example, at least two VAL homologues, Table 1), but as the genome sequencing of this parasite is undertaken, this deficiency should soon be addressed.

*H. polygyrus* is closely related to *N. brasiliensis* but is able to establish chronic infections in mice. Immunosuppressive properties of *H. polygyrus* ES (HES) were first shown by Pritchard and colleagues on KLH-specific bystander responses *in vitro* [64]. More recently, a single HES fraction was reported to inhibit T cell proliferation and macrophage nitric oxide production [65]. HES treatment of DCs ablates IL-12p70 responsiveness to TLR agonists such as LPS [66]. Furthermore, HES-exposed DCs can induce differentiation of IL-10-producing CD4^+^ Tregs, which suppress bystander T cell proliferation [66]. One candidate immunomodulator is calreticulin, secreted by tissue-phase intestinal larvae, which can induce Th2 differentiation [67]. We have also established that at least six homologues of VAL are secreted by the adult worm (Table 1), as well as a TGF-β-like ligand which induces functional, suppressive Tregs from naive precursors (see Section 4.4 below).

### 3.4 Human and canine hookworms: *Ancylostoma caninum* and *Necator americanus*

Hookworm research has focussed on both the infective L3 stage, as a vaccine target, and on the blood-feeding adult worms. *A. caninum* L3 release the VAL homologue *Ancylostoma* secreted protein (ASP) [68], and a similar antigen from the human hookworm *N. americanus* is now in a vaccine trial [69]. ASPs are also abundant in adult *A. caninum* ES [70], together with proteases which play a role as anti-coagulants and in digestion of blood contents [71]. *A. caninum* adult-secreted mediators include a fatty acid/retinol binding protein [72], and a tissue inhibitor of metalloprotease [73] while an adult *N. americanus* protein binds to human NK cells, resulting in IFN-γ production [74]. Finally, *A. caninum* ES can reduce TNBS-induced intestinal inflammation, demonstrating its immunomodulatory potential [75].

### 3.5 Trichostrongyles of ruminants: *Haemonchus contortus* and related species

*H. contortus* is a trichostrongyle nematode and one of the most prevalent helminth parasites, distributed in ruminant livestock worldwide. Vaccination of sheep with *H. contortus* adult ES proteins induces significant protection (>70%) against challenge [76]; the major antigens are Hc15, and Hc24, the latter being a VAL homologue [77]. Proteomic analysis of the ES [78] indicates both Hc24 and a further VAL homologue Hc40 are expressed as numerous isoforms; galectins (GALs) are also prominent. Other intestinal nematodes of livestock, very closely related to *H. contortus,* secrete a similar GAL/VAL-dominated suite of ES proteins including *Cooperia* spp. [79], *Ostertagia ostertagi* [80], and *Teladorsagia circumcincta* [81].

### 3.6 *Toxocara canisand Trichinella spiralis*

*T. canis* is a parasite which, in its larval form, can infect a wide variety of hosts, causing visceral larva migrans in humans. Larval TES is type-2 stimulating [82] and comprises a relatively simple set of glycoproteins which is dominated by three gene families [83]. Most ES proteins match a small transcriptomic dataset [84], reflecting the secretion of a small number of relatively abundant proteins, including two C-type lectins [85,86] and three mucins [87,88]. The latter carry abundant *O*-linked glycans, similar in structure to mammalian blood group H [89], which are the target of dominant IgM antibodies in infected hosts [90].

*T. spiralis* (the pork worm) can also infect a broad host range, and ES antigens from this parasite were among the first to be characterised by biosynthetic labelling [91]. An intriguing set of functional properties have been discovered in ES, including the only known secreted protein kinase [92], a 5′-nucleotidase [93], macrophage migration inhibitory factor [94], and a prosaposin [95]. A *T. spiralis* nucleoside diphosphate kinase is secreted [96] and a similar product reported in ES from other nematodes [78,81]. Detailed proteomic analyses of the muscle-stage (infective) larvae have been undertaken [97,98].

### 3.7 *Taenia* and *Echinococcus*

Larval forms of cestode Taeniid tapeworms cause cystercercosis in humans; a model of this disease is *T. crassiceps* in mice, in which larval parasites in the peritoneal cavity can multiply asexually, accompanied by suppression of Th1 responses [99]. Larval ES products suppress *in vitro* T cell responses [100], although individual components of the secreted material were not identified. Additionally, larval ES contains a functional mimic of host IFN-γ, but the role of this protein in immunoregulation is unclear [101]. Hydatid cysts, surrounding metacestodes of *Echinococcus granulosus*, are considered to comprise both host proteins and parasite secretions: prominent among the latter are the antigen B family which is implicated in Th2 induction and is reported to inhibit neutrophil migration [102].

## 4 Immunomodulatory molecules from helminths

### 4.1 Alpha to omega of schistosome Th2 induction

The schistosome-secreted proteins alpha-1 and omega-1 promote Th2 differentiation. Alpha-1, released by schistosome eggs [28], induces IL-4 release and degranulation by human and mouse basophils, thereby initiating a Th2 environment [103,104]. Also named IL-4-inducing principle of schistosome eggs (IPSE), alpha-1 is a dimer that binds and cross-links surface IgE on basophils, in an antigen-independent manner. IPSE has also been shown to function as a chemokine binding protein, which by sequestering ligands, can prevent chemokine-mediated recruitment of inflammatory cells such as neutrophils [105]. Neutralisation of IPSE, using polyclonal sera, leads to increased egg-induced inflammation, directly implicating IPSE in the modulation of egg granulomatous responses. Omega-1 is a ribonuclease abundantly secreted by eggs [106] which is hypothesised to stimulate the immune response necessary for egg transit across host tissues, allowing excretion. Supporting this, recent evidence indicates omega-1 can directly induce Th2 responses (M. Mohrs, M. Yazdanbakhsh and G. Schramm personal communication).

### 4.2 ES-62 and phosphorylcholine inhibition of immune cell signalling

Phosphorycholine is a small hapten-like moiety present in secretions of many helminths. ES-62 is the leucine aminopeptidase secreted by *A. viteae*, which is heavily conjugated with phosphorylcholine and represents the dominant ES product of adult worms of this species [107]. Through PC modifications, ES-62 can inhibit the proliferation of CD4^+^ T cells and conventional B2 cells *in vivo*, and reduces CD4^+^ cell IL-4 and IFN-γ production [108,109]. Conversely, ES-62 promotes proliferation and IL-10 production by peritoneal B1 cells [110]. Antigen-presenting cells are also targeted, as ES-62 pulsed bone marrow-derived DCs drive Th2 differentiation *in vitro* [111], and pre-treatment of DC and macrophages with ES-62 inhibits their ability to produce IL-12p70 in response to LPS [112]. Inhibition of pro-inflammatory Th1 responses occurs as ES-62 interacts with toll-like receptor (TLR) 4 through its PC residues [113], and in mast cells TLR4 binding results in the sequestration and degradation of intracellular PKCa, thereby inhibiting degranulation and release of inflammatory mediators [114]. ES-62 also protects mice against collagen-induced arthritis [115].

Notably, in *B. malayi* PC is not found on the ES-62 homologue (LAP), but on another secretory protein, *N-*acetylglucosaminyltransferase [29]. In the rodent filarial parasite *Litomosoides sigmodontis*, the major ES product is modified with DMAE (dimethylaminoethanol) [116], which contains one less methyl group than PC, giving rise to suggestions that DMAE may function immunologically in a manner similar to PC [117].

### 4.3 Glycans and lipid molecules—connecting with DCs?

Helminth ES preparations are generally rich in glycoproteins and lipids, leading to many potential interactions with innate pattern-recognition receptors, such as TLRs and C-type lectins on host DCs. Blood group-like glycans from *T. canis* bind the lectin DC-SIGN, hypothesised to favour immune regulation [90]. Schistosome glycoproteins show extensive glycosylation [32], including Lewis^X^ motifs that trigger Th2 responses *in vivo* through TLR4 ligation [118]. The consequences of glycan-dependent stimulation include granuloma development *in vivo* [119]. Additionally, macrophage stimulation by schistosome larval secretions is dependent on carbohydrates [120]. Helminth lipids have also been implicated in immune modulation; schistosome phosphatidylserine (PS) induces DCs to polarise IL-4/IL-10-producing T cells. In contrast, schistosome lyso-PS, containing only a single acyl chain, conditions DCs to induce IL-10 secreting regulatory T cells, thus swaying the immune system away from a protective Th2 response [121].

### 4.4 Cytokine homologues—on the host's home turf

It is now clear that certain highly conserved cytokine gene families are present in helminths, and that their products can ligate receptors on mammalian immune cells. For example, *B. malayi* and *A. ceylanicum* express homologues of the mammalian cytokine macrophage migration inhibitory factor (MIF) [122]. Mammalian MIF is considered to be pro-inflammatory, playing a key role for example in septic shock. Perhaps surprisingly, nematode MIF homologues mimic host MIF by induction of pro-inflammatory cytokines [50,123,124]. However, we have recently found that *Brugia* MIF synergises with IL-4 to induce the development of fully suppressive alternatively activated macrophages *in vitro* [125], to a level beyond that observed for IL-4 alone [126]. One pathway for this effect may be through the induction by MIF of IL-4R expression on macrophages [125], thereby amplifying the potency of IL-4 itself. Thus, in a Th2 environment, MIF may prevent the classical, pro-inflammatory, activation of macrophages.

Worms also express members of the TGF-β and TGF-β receptor superfamilies. *B. malayi* adults secrete TGH-2, a homologue of host TGF-β and of the *C. elegans* developmental protein, DAF-7 [35]. Recombinant TGH-2 can bind to the mammalian TGF-β receptor, suggesting it may promote the generation of regulatory T cells [127], as has been found for mammalian TGF-β. However, TGH-2 is secreted at very low levels, below the limit of detection for proteomics, and it is unclear whether this is sufficient for bioactivity [29,30]. In contrast, a *H. polygyrus* TGF-β mimic is able to directly induce Foxp3^+^ expression in activated T cells, implying a key role in parasite immune avoidance (Grainger et al., submitted for publication). Parasite TGF-β homologues also have non-immune roles, and one such *S. mansoni* protein is involved in egg development [128].

### 4.5 C-type lectins and galectins—targetting mammalian glycans?

Lectins are carbohydrate binding proteins, and host C-type lectins and galectins are involved in a variety of immune processes, such as antigen uptake and presentation, cell adhesion, apoptosis and T cell polarisation [129]. C-type lectins (C-TLs) are particularly abundant in the secretions of *T. canis* [85,86] and those of hookworms [70]. The biological roles of parasite C-TLs are unclear, but two *T. canis* C-TLs (TES-32 and TES-70) show greater homology to mammalian proteins such as CD23 (low affinity IgE receptor) and macrophage mannose receptor, than to any *C. elegans* protein [86]. Furthermore, TES-70 is able to bind mammalian carbohydrates in a calcium-dependent manner [85] suggesting a role in immune evasion by e.g. inhibiting the migration of host cells. Alternatively, parasite C-TLs may bind to and mask worm carbohydrates from host immune cells. Additionally, nematode C-TLs have roles unconnected with immune evasion. The acquisition of symbiotic bacteria by the marine nematode *Laxus oneistus* requires its secretion of a C-TL [130], while a non-secretory C-TL from *A. ceylanicum*, specifically expressed by sperm cells, has a putative role in nematode reproduction [131]. Secreted galectins are more apparent in other species such as *H. contortus* [132] and particularly *B. malayi* [29]. A recombinant *Brugia* galectin, *Bm*-GAL-1, is able to bind to host immune cells in a carbohydrate dependent manner (J.P.H. unpublished observations), but does not share the eosinophil chemoattractant properties reported for a *H. contortus* galectin [133].

### 4.6 Protease inhibitors—blocking innate cell functions

Two highly expressed sets of protease inhibitors are the cystatins and the serpins, each with proposed immunomodulatory roles. Cystatins (cysteine protease inhibitors) from *A. viteae*, *B. malayi*, *O. volvulus* and *N. brasiliensis* act as immunomodulators, through at least two mechanisms [134,135]. Firstly, they inhibit cysteine proteases (cathepsins and aspartyl endopeptidase) required for host APC antigen processing and presentation, so leading to reduced T cell priming [136,137]. Secondly, they elicit the immunosuppressive cytokine IL-10, leading to a reduction in costimulatory molecule expression by APCs, and the direct inhibition of T cell proliferation [138]. The immunomodulatory potential of parasite cystatins is also evident *in vivo*, in inhibition of both allergic lung inflammation and colitis, mediated by Tregs and IL-10-producing macrophages [139].

The serpins are serine protease inhibitors [140], and one member of this family, SPN-2, is the major mRNA and secreted protein product [30,141] of *B. malayi* microfilariae. The function of SPN-2 is disputed; in collaboration with a leading serpin laboratory we reported specific inhibition of the neutrophil proteinases cathepsin G and neutrophil elastase, and no activity against a range of other enzymes such as pancreatic chymotrypsin and coagulation factors [53]. However, an independent group reported that recombinant protein was devoid of inhibitory activity [142]. Irrespective of direct anti-enzymatic activity, SPN-2 stands out as unusual because of its ability to stimulate a Th1 response in mice, corresponding to the ability of live microfilariae to drive this type of immune response [141].

### 4.7 Antioxidants and acetylcholinesterases

Production of reactive oxygen species (oxygen radicals, superoxide, and hydrogen peroxide) by phagocytes is a primary pathway of immune attack against parasites. Correspondingly, most parasites express high levels of antioxidants, including superoxide dismutases (SODs), catalases, glutathione and thioredoxin peroxidases, and peroxiredoxins. Secreted helminth antioxidant enzymes include *B. malayi* glutathione peroxidase [29] and SOD [143], and thioredoxin peroxidase from *F. hepatica* [46]. In the latter case, the enzyme is also responsible for inducing alternatively activated macrophages [46].

Acetylcholinesterase (AChE) breaks down the neurotransmitter acetylcholine in order to terminate neuronal signals, and is active in the neuromuscular system of helminths. AChE has been identified in the ES of many gut-dwelling nematodes, including *H. polygyrus* [144], *N. brasiliensis* [145], the lungworm *Dictyocaulus viviparus* [146], and adult *B. malayi* [147]. It has been proposed that their secretion may also hydrolyse acetylcholine from the enteric nervous system of the host [148]. Since acetylcholine-mediated signalling stimulates intestinal chloride and mucus production, AChEs may prevent fluid increases in the gut that promote parasite clearance. Finally, another *N. brasiliensis* secreted enzyme is platelet activating factor (PAF) hydrolase, which is likely to act in an anti-inflammatory capacity on the platelet population [149].

### 4.8 Venom allergen/ASP-like (VAL) homologues

In 1996, the Hotez laboratory described the *A.* *caninum* secreted protein, ASP [68], the first of an enigmatic gene family expressed across a wide variety of parasitic helminths, including human hookworm [150] filarial nematodes [30,151], trichostrongylids such as *H. contortus* [77,78], schistosomes [28,41,152], as well as free-living *C. elegans* [153]. We have termed this the Venom allergen/ASP-Like (VAL) gene family [151]. Alongside mammalian cysteine-rich sperm proteins (CRISPs), insect venom allergens and plant pathogenesis family-1 (PR-1) proteins, VAL proteins are members of the SCP (sperm coating protein)-1 superfamily. Despite sequence similarity, no coherent function for this protein family has been demonstrated. An *A. caninum* SCP-1 protein, neutrophil inhibitory factor (NIF), binds the host integrin CR3 (CD11b/CD18) and is able to inhibit neutrophil function, including oxidative burst [154,155].

The crystal structure of *N. americanus* ASP-2 reveals a charge segregation reminiscent of mammalian chemokines, suggesting that this protein may be a ligand or antagonist for G-protein coupled receptors such as the chemokine receptors [156]. Consistent with this prediction, *Na*-ASP-2 has recently been shown to induce neutrophil chemotaxis *in vitro* and *in vivo* [157], but it remains uncertain if this is a widespread property of VAL homologues. An alternative possibility is that the SCP-1 domain provides a stable structural backbone, allowing the non-conserved regions of the different VAL proteins to carry out numerous different roles [70]. Even if this were the case, the prominence of VAL products in most helminth secretions is highly suggestive of an important role in modifying host immunity.

### 4.9 Novel proteins

Helminths secrete numerous products lacking discernable sequence similarity to known proteins. Examples include the filarial ALT-1 and ALT-2 proteins which are highly abundant in the infective larval stage [158,159]. One route to determine the function of these proteins has been by heterologous expression in *Leishmania* parasites, studying changes in immune responsiveness resulting from filarial gene expression. *L. mexicana* parasites expressing *B. malayi* ALT-1 or ALT-2 were found to reach significantly higher levels of infection in macrophages *in vitro*, inhibiting killing mechanisms, and were more virulent *in vivo* [160]. Cells harboring transgenic parasites upregulated SOCS-1, an inhibitor of IFN-γ signalling, suggesting that the ALT proteins impair Th1 responsiveness known to be required for immunity in this system [160].

A *B. malayi* polyprotein “ladder” gp15/400 represents another unusual filarial immunomodulator. Adults synthesise this protein as a large 400-kDa precursor, subsequently processed into secreted 15-kDa subunits [161]. Released subunits can bind host retinoids [162], a property that may be shared with another family of secreted proteins, the transthyretin-like proteins [29]. Given that retinoic acid can synergise with TGF-β to induce Foxp3^+^ Tregs [163], it is possible that such proteins could enhance vitamin A uptake by host tissues to favour conversion to RA and thus enhance Foxp3^+^ Treg induction. The homologue of gp15/400 from *Dirofilaria immitis*, a filarial worm of dogs, stimulates mouse B cell synthesis of IgE through direct binding to CD40 [164] and can also inhibit insulin-dependent diabetes in mice [165].

## 5 Conclusion

**Fig. 2:**
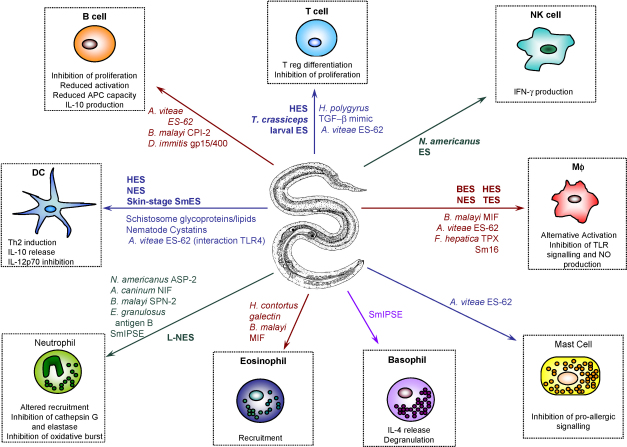
Mechanisms of immune modulation by helminth ES products (in bold) and defined molecules (in plain type) discussed in the text. *Abbreviations*: **APC**, antigen presenting cell; **ASP**, *Ancylostoma* secreted protein; **BES**, *B. malayi* ES; **CPI**, cysteine proteinase inhibitor (cystatin); **HES**, *H. polygyrus* ES; **IPSE**, IL-4-inducing principle of schistosome eggs; **L-NES**, larval *N. brasiliensis* ES; **MIF**, macrophage migration inhibitory factor; **NES**, adult *N. brasiliensis* ES; **NIF**, neutrophil inhibitory factor; **Sm**, *Schistosoma mansoni*; **SPN**, serine proteinase inhibitor (serpin); **TLR**, toll-like receptor; **TGF**, transforming growth factor; **TES**, *T. canis* ES.

The systematic analysis of ES products, which has become possible through the combination of proteomics and genomics, is now providing us with a comprehensive catalogue of potential immunomodulators, each pointing the way towards critical interactions between parasites and the host immune system (Fig. 2). Many parasites have targeted similar host pathways, particularly within innate immunity, but the detailed mechanisms differ because each helminth species has evolved its own strategy to confound host defences. Identification of these specific mechanisms may allow the development of neutralising vaccines that promote worm clearance. Moreover, the striking protective effect of helminth infections, in many contexts, against immunopathological disorders [9,115,166], and the introduction of therapeutic helminth infections [8], sets an urgent agenda to replace live parasite therapy with non-living parasite products. The recent advances in ES are likely to have already identified the candidates, and as we have described here, provided exciting early data on the ability of these proteins to modulate host immunity.

## Note added in proof

Bennuru et al. [170] have performed a comprehensive proteomic analysis of the ES proteins from L3, L3 to L4 moult, MF and adult *B. malayi*, resulting in the identification of 852 proteins. This supports the previous studies [29,30], and additionally shows the abundant secretion of ALT family members by larval parasites, as well as the release of trace amounts of *Wolbachia endosymbiont* proteins. Robinson et al. [171] have also made available an in-depth proteomic analysis of the *F. hepatica* secretome based on new transcriptomic data.

## Figures and Tables

**Table 1:** Proteomic analyses of helminth secretions.

Species	Stage/niche	Proteins identified	Prominent proteins	Reference	Notes
*Ancylostoma caninum*	Adult/duodenum	105	ASPs (VALs)	[70]	Over 30 different VAL homologues present
			C-type lectins and galectins, proteases		
*Brugia malayi*	Adult, male and female/lymphatics	80	Triose phosphate isomerase	[29,30]	GlcNAcT, but not LAP, bears PC
		193	Galectin, GlcNAcT		
			LAP, NPA, MIF-1		
	Microfilaria/blood	76	Serpin-2	[30]	
			PEBP, Bm-R1		
*Haemonchus contortus*	Adult/abomosum	107	VALs, proteases, gut proteins	[78]	Multiple VALs
*Heligmosomoides polygyrus*	Adult/duodenum	44	VALs, proteases, NPA, acetylcholinesterase	Harcus unpublished^a^	Multiple VALs
*Nippostrongylus brasiliensis*	Adult/duodenum	3	VALs, globin	Harcus unpublished^a^	
*Ostertagia ostertagi*	Adult/abomosum	2	VALs	[80]	
*Schistosoma mansoni*	Larva (schistosomula)/skin and lung	16	Cercarial elastase	[40-42]	
		82	Metalloproteinase		
			VALs, Sm16		
	Adult, gut contents/blood	8	Antioxidants, cystatin	[167]	Gut contents likely to be released as “ES”
			FABP, immunophilin		
	Egg/GI tract	188	IPSE (alpha-1), omega-1	[28]	
			VALs, aldolase, enolase		
*Teladorsagia circumcincta*	Larva (L3/L4) and adult/abomosum	15 larval	VALs, proteases, TPX	[81]	
		13 adult		[168]	
*Toxocara canis*	Larva (L2)/tissues	8	Mucins, C-type lectins, PEBP	[88]	
				Harcus unpublished^a^	
*Trichinella spiralis*	Muscle-stage (L1) larva	43	Cystatin, 5′ nucleotidase	[97]	
			Galectin, proteases		
Stages or species not parasitic to vertebrates					
*Fasciola hepatica*	Mollusc-dwelling larva	8	Antioxidants (SOD, TRX)	[47]	
*Meloidogyne incognita*	Plant parasitic	486	Heat shock proteins	[51]	Interesting overlap with *B. malayi* ES
			Glycolytic enzymes		
*Schistosoma mansoni*	Sporocyst (snail dwelling)	7	Antioxidants (SOD, GST)	[169]	
			Glycolytic enzymes (aldolase, enolase, triose phosphate isomerase)		

*Abbreviations*: ASP, ancylostoma secreted protein; FABP, fatty acid binding protein; GlcNAcT, *N*-acetylglucosaminyltransferas; GST, glutathione-*S*-transferase; IPSE, IL-4 inducing principle of schistosome eggs; LAP, leucyl aminopeptidase; MIF, macrophage migration inhibitory factor homologue; NPA, nematode polyprotein allergen; PC, phosphorylcholine; PEBP, phosphatidylethanolamine binding protein; SOD, superoxide dismutase; TPX, thioredoxin peroxidase; TRX, thioredoxin; VAL, venom allergen/Ancylostoma secreted protein-like proteins; BmR1 and Sm16 are non-acronymic designations.

a Harcus, Y., Hewitson, J., Curwen, R., Dowie, A., Ashton, P., Wilson, R.A. and Maizels, R.M., manuscript in preparation.
